# Characterizing the Soil Microbial Community Associated with the Fungal Pathogen *Coccidioides immitis*

**DOI:** 10.3390/jof11040309

**Published:** 2025-04-14

**Authors:** Molly Radosevich, Jennifer Head, Lisa Couper, Amanda Weaver, Simon Camponuri, Liliam Montoya, John W. Taylor, Justin Remais

**Affiliations:** 1 Environmental Health Sciences, University of California Berkeley, Berkeley, CA 94720, USA; molly_radosevich@berkeley.edu (M.R.); simonkcampo@berkeley.edu (S.C.); 2 Department of Epidemiology, University of Michigan, Ann Arbor, MI 48109, USA; jrhead@umich.edu; 3 Institute of Global Change Biology, University of Michigan, Ann Arbor, MI 48109, USA; 4 Plant and Microbial Biology, University of California Berkeley, Berkeley, CA 94720, USA

**Keywords:** *Coccidioides*, coccidioidomycosis, Valley fever, soil, microbial communities, pathogen

## Abstract

Coccidioidomycosis is a fungal disease affecting humans and other mammals caused by environmental pathogens of the genus *Coccidioides*. Human exposure to the pathogen occurs via inhalation of spores aerosolized from soil. Thus, understanding the ecological factors that shape the distribution of *Coccidioides* in soils is important for minimizing the risk of human exposure, though this task remains challenging due to the pathogen’s highly variable spatial distribution. Here, we examined the associations between the soil microbial community and *Coccidioides immitis*’ presence within the Carrizo Plain National Monument, a minimally disturbed grassland ecosystem, and the site of a longitudinal study examining the effects of rodents and their burrows on *C. immitis*’ presence in soils. Using internal transcribed spacer 2 (ITS2) and 16S amplicon sequencing to characterize the soil fungal and bacterial communities, we found over 30 fungal species, including several other members of the Onygenales order, that co-occurred with *C. immitis* more frequently than would be expected by chance. *Coccidioides*-positive samples were significantly higher in fungal and bacterial diversity than negative samples, an association partly driven by higher *Coccidioides* presence within rodent burrows compared to surface soils. Soil source (i.e., rodent burrow versus surface soil) explained the largest amount of variation in bacterial and fungal community diversity and composition, with soils collected from rodent burrows having higher fungal and bacterial diversity than those collected from adjacent surface soils. While prior evidence is mixed regarding the relationship between the presence of *Coccidioides* and microbial diversity, we find that favorable microhabitats, such as rodent burrows, lead to a positive association between soil microbial diversity and *Coccidioides* presence, particularly in otherwise resource-limited natural environments.

## 1. Introduction

*Coccidioides*, a genus of soil-dwelling, rodent-associated pathogenic fungi within the *Onygenaceae* family that cause coccidioidomycosis (also known as Valley fever), is among the priority fungal pathogens of concern identified by the World Health Organization (WHO) [1,2,3]. There is currently no available vaccine to prevent coccidioidomycosis, making reducing exposure to the pathogen the primary method of disease prevention. However, identifying areas of high risk for human exposure remains challenging as the pathogen’s presence in the environment varies widely over fine spatial scales. While coccidioidomycosis incidence rates in California and Arizona have been linked to seasonal trends in temperature and precipitation and interannual drought [4,5,6], the ecological factors driving the patchy distribution of environmental *Coccidioides* populations remain poorly understood [7,8]. As such, a better understanding of these factors is critical for identifying point sources of pathogen exposure risk.

**Figure 1:**
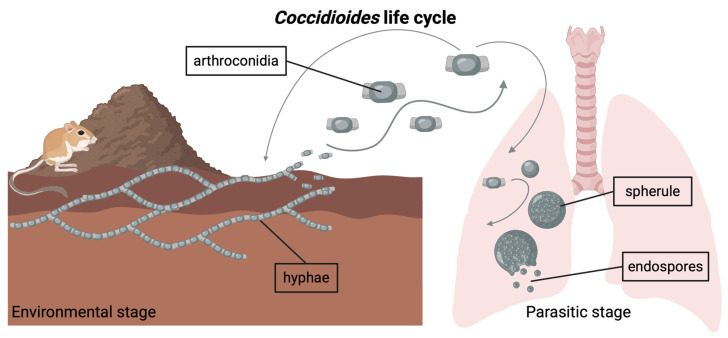
*Coccidioides* is a genus of dimorphic fungi with an environmental stage and a parasitic stage. In its environmental stage, the fungus grows in the soil as long chains of branching hyphae. Mature hyphae generate arthroconidia, which can become airborne if the soil is disturbed. Inhalation of arthroconidia can lead to infection with *Coccidioides*. Inside the host lungs, the arthroconidia mature into spherules, inside which endospores form. Eventually, the spherules rupture and release the endospores. Note: Graphic components are not drawn to scale.

Both *Coccidioides* species (i.e., *C. immitis*, *C. posadasii*) have a dimorphic life cycle (Figure 1) and are found in arid regions of the Americas [9]. In the soil, *Coccidioides* grows as a network of branching hyphae, potentially obtaining carbon and nutrients from the bodies of dead rodents and other sources of animal keratin shed into burrows and the surrounding soil [9,10,11]. As the hyphae mature, they produce chains of asexual spores known as arthroconidia, which become airborne when the soil is disturbed by excavation or wind erosion [12,13]. When a mammal inhales these spores and is unable to control the infection, the fungus initiates the parasitic phase of its life cycle inside the host lungs. Symptomatic infections occur in ~40% of human cases [14]. In rare cases, the fungus can disseminate beyond the lungs to other parts of the body. Upon mammalian host death in the environment, *Coccidioides* may be released from host immunological control, where it is subsequently hypothesized to utilize the carcass as a source of nutrients [1,9,10].

In regions known to harbor *Coccidioides*, its spatiotemporal distribution in the soil is sporadic and uneven [7,15]. In some cases, *Coccidioides*-positive sites are identified only in the wake of a coccidioidomycosis outbreak linked to a specific geographic area and timeframe [16,17,18,19,20]. However, it is not uncommon, even in studies sampling in putatively positive areas based on epidemiologic data, for the fungus to be detected in few or none of the samples drawn from soils (e.g., <10%) [21]. Prior limitations in the molecular detection methods for *Coccidioides* may have contributed to the observed unevenness in the soil, but recent advancements in the sensitivity and specificity of PCR assays for the pathogen have minimized this challenge, lowering the limit of detection to as low as <15 target DNA copies per reaction [22].

The presence and abundance of wild populations of mammalian host species may play an important role in maintaining environmental *Coccidioides* spp. populations [10,23,24]. As stated by the endozoan, small-mammal reservoir hypothesis, *Coccidioides* spp. are posited to persist in the soil primarily through infection of rodents and other small mammals [10]. Several studies have detected *Coccidioides* inside rodent burrows more often than in the surrounding soil, with positivity rates of ~20–30% in burrows and ~4–13% outside of burrows [24,25]. Further, recent work found that rodent burrow creation mediates the effect of rodents on the presence of *Coccidioides*, with 73.7% of the association between rodents and *Coccidioides* attributable to the creation of burrows [25].

Despite these findings, our understanding of how the relationships between rodents, burrows, and *Coccidioides* impact and are impacted by the underlying soil microbial community remains unexplored. As antifungal activity is relatively common amongst soil bacteria [26,27], a few studies have isolated naturally occurring microbes from the soil at *Coccidioides*-positive sites that inhibit the pathogen’s growth on plates, consistent with the suggestion that *Coccidioides* is a poor competitor against other soil microorganisms [28,29,30]. However, the impact of these antagonistic organisms in natural, ecological settings is unclear. The soil microbial community of which *Coccidioides* spp. is a constituent has not been extensively characterized, though one study found an increase in alpha diversity in soils containing more *Coccidioides* [31], while another found no statistically significant association between *Coccidioides* presence and overall fungal community composition [32]. Thus, the relationship between the soil microbial community and *Coccidioides* presence remains an important open question. Determining how the soil microbial community may influence *Coccidioides*’ ecology and structure *Coccidioides* populations in the soil can inform recommendations on minimizing human exposure risk to the pathogen, including through the incorporation of ecological diversity measures and indicator taxa into pathogen distribution models and the identification of potential biocontrol agents.

Our study represents the most comprehensive characterization of the natural soil microbial community associated with environmental *Coccidioides* spp. populations to date. Herein, we investigated the relationship between soil bacterial and fungal communities and *Coccidioides* presence, as well as whether and how this relationship may be mediated by the soil microhabitat. We leveraged a 14-year ecological experiment on rodent herbivory in the Carrizo Plain National Monument in California—a region known to harbor *Coccidioides immitis* [33]—to investigate the relationship between soil microbial community dynamics and the presence of *C. immitis* in replicated plots where rodents had or had not been excluded. We tested the null hypotheses that there would be no significant differences in soil microbial (fungal and bacterial) community diversity or composition based on the following variables: *C. immitis* status (whether a sample tested positive for the pathogen via qPCR or not), sample source (whether a sample was collected from inside a rodent burrow or from the surrounding ground surface), and rodent status (whether rodents were present in or excluded from the local soil sampling environment). Additionally, we tested the null hypothesis that no individual fungal or bacterial taxa would co-occur with *C. immitis* more often or less often than expected by chance.

To investigate our hypotheses, we collected soil samples from the ground surface and from burrows in plots where rodents have been present or have been excluded and analyzed the soil microbial communities. This design allowed us to disentangle the influence of rodent presence and the burrow microhabitat on soil microbial populations and *Coccidioides* presence, which is a key open question [25]. Finally, we investigated whether specific fungal or bacterial taxa have positive or negative co-occurrence patterns with *C. immitis*. Our study highlights the importance of continued environmental sampling and investigation of *Coccidioides* in its natural environment, with implications for identifying sources of *Coccidioides* in the soil, understanding microbial community dynamics in relation to environmental *Coccidioides* populations, and modeling the pathogen’s distribution.

## 2. Materials and Methods

### 2.1. Soil Collection

**Figure 2:**
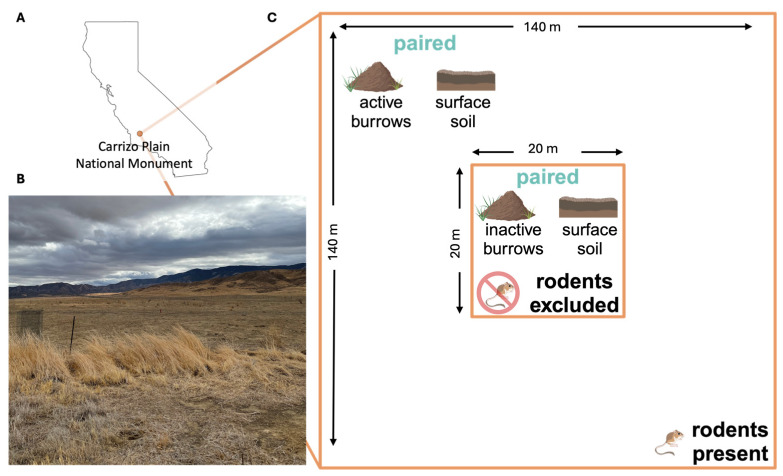
(**A**) Location of the Carrizo Plain National Monument (orange point) in California. (**B**) A photograph from the field site: a grassland plain bordered to the northeast by the Temblor Range and the southwest by the Caliente Range, taken in October 2021. (**C**) Schematic of one sampling plot, comprising an inner zone (center; 20 m × 20 m) where rodents are excluded by a physical barrier (exclosure), and surrounded by an outer zone (140 m × 140 m), where rodents are present (Note: figure is not to scale). Five replicate precinct soil samples are collected from inactive burrows within the rodent exclosure and paired with five adjacent soil samples collected from the ground surface within the exclosure (see Materials and Methods Section 2.1). Five paired precinct samples and five adjacent surface soil samples are also collected in the surrounding zone where rodents are present.

We collected soil samples from study sites within the Carrizo Plain National Monument in eastern San Luis Obispo County, California (Figure 2A,B), an ecosystem characterized by extensive rodent burrow systems, where an ongoing, long-term experiment is examining the impacts of drought, livestock management, *Dipodomys ingens* (giant kangaroo rat) activity, and the presence of other wildlife on ecological communities [34,35]. Samples were collected across two pastures in the monument. One pasture experiences periodic cattle grazing and is dominated by several exotic annual grasses, while the other pasture has never been grazed and is a dominated by a single native perennial grass species [34]. Within each pasture, we collected samples from 8 plots, each measuring 140 × 140 m (m), and each containing, at its center, a 20 × 20 m rodent exclosure fence established in 2007 (Figure 2C) [34]. The rodent exclosure fence extends 61 cm (cm) into the soil and 91 cm above the ground surface. At the time of establishment, and monthly thereafter, any rodents found inside the fence were relocated [34]. At each plot, outside of the rodent exclosure where there is active rodent presence, we randomly selected one rodent burrow system (a precinct) and collected replicate precinct samples from inside the entrances of five burrows; these samples were paired with five replicate topsoil samples collected starting at two meters away from the nearest burrow entrance and subsequently at one-meter intervals from a depth of 10 cm below the ground surface. Inside the exclosures, where rodent access has been prevented since 2007, we obtained five precinct samples and five topsoil samples following the same procedure used outside the exclosure. All samples analyzed were collected in April 2021.

### 2.2. Soil DNA Extraction

Soil samples were kept at room temperature until DNA extraction, as is standard protocol for the detection of *Coccidioides* in soils [11,36,37]. While cold storage is often used in microbial community analysis, evidence suggests that DNA-based microbial community composition and diversity analyses of soil are relatively robust to storage conditions, including storage at room temperature for 14 days after collection [38,39,40,41,42], demonstrating the utility of our samples for microbial community analysis even under ambient storage conditions in the laboratory. We extracted genomic DNA from all soil samples using the DNeasy PowerSoil Pro Kit (QIAGEN, Hilden, Germany), with modifications to the protocol in accordance with Biosafety Level 2+ control measures (see Supplementary Text S1).

### 2.3. Quantitative PCR to Detect Coccidioides spp.

After DNA extraction and quantification via the Qubit^TM^ dsDNA Quantification High Sensitivity Assay Kit (Thermo Fisher Scientific, Waltham, MA, USA), we diluted samples to 12 ng/µL in preparation for qPCR (i.e., to minimize PCR inhibition occurring at higher DNA concentrations as in Bowers et al. (2019)) [22]. We conducted qPCR in 96-well plates following the CocciENV assay (see Supplementary Text S2) and processed all samples in quadruplicate plate wells [22,43]. Samples were considered positive for *Coccidioides* spp. if at least 3 out of 4 wells had a Ct value below 40 [22,32].

### 2.4. ITS2 and 16S Amplicon Sequencing

After assessing the presence of *Coccidioides* in each sample, we prepared samples for PCR amplification and pooling into both internal transcribed spacer 2 (ITS2) libraries (fungi) and 16S libraries (bacteria). ITS2 is a variable region of nuclear ribosomal DNA that allows for discrimination between species of fungi [44,45,46]. Similarly, the 16S gene region encodes for ribosomal RNA and is commonly used to discriminate between genera or families of bacteria [47]. We used 5.8S-Fun (5′-AACTTTYRRCAAYGGATCWCT-3′) and ITS4-Fun (5′-AGCCTCCGCTTATTGATATGCTTAART-3′) primers for ITS2 sequence amplification [48,49], and 341F (5′-CCTACGGGNBGCASCAG-3′) and 785R (5′-GACTACNVGGGTATCTAATCC-3′) primers for amplification of the 16S V3–V4 gene region [50]. We set a target sample size of 300 samples to obtain sufficient sequencing depth (averaging 100,000 reads per sample). Positive controls (*Neurospora crassa* genomic DNA from the Fungal Genetics Stock Center (Manhattan, KS, USA) for ITS2; ZymoBIOMICS Microbial Community Standard DNA (Zymo Research, Irvine, CA, USA) for 16S) and negative controls (water) were run on each PCR plate to confirm amplification of the intended regions and no contamination of samples. Libraries were sent to the Vincent J. Coates Genomic Sequencing Laboratory (QB3 Genomics, UC Berkeley, Berkeley, CA, USA, RRID:SCR_022170) for fragment analysis and sequencing using the Illumina MiSeq v3 300 PE kit (Illumina, Inc., San Diego, CA, USA). The PCR amplification protocol was based on a methodology detailed elsewhere [49,50]. The sequencing data generated through this project are available through the National Center for Biotechnology Information Sequence Read Archive at accession numbers PRJNA1201328 and PRJNA1201319.

### 2.5. Amplicon Sequence Processing

After sequencing and demultiplexing, we processed the sequences through the QIIME2 pipeline [51]. We used Cutadapt to trim primers out of all sequences [52] and used a minimum quality score cutoff of 25 to identify where to truncate all sequences before downstream analysis. We then processed the sequences in DADA2 to truncate and merge forward and reverse sequences, using the consensus method to exclude chimeras [53]. Finally, all merged amplicon sequence variants (ASVs) were assigned taxonomy using the UNITE database (version 8.3) for fungi (97% similarity) [54,55] and the SILVA database for bacteria (99% similarity) [56]. Fungal taxa assigned “unspecified” (indicating sequences that could not be resolved to a species) or “unidentified” (indicating sequences not annotated within the reference database) were removed from further analysis as these categories may group different species together under one classification. Similarly, bacterial taxa assigned “unidentified”, “Unknown”, or “uncultured” were removed from downstream analysis.

### 2.6. Statistical Analyses

Using fungal and bacterial DNA sequences, we estimated microbial community diversity and composition via the Shannon index and Bray–Curtis dissimilarity to estimate alpha and beta diversity, respectively [57,58]. We also estimated co-occurrence patterns with *Coccidioides* via checkerboard score analysis [59]. For all analyses, we analyzed ITS2 and 16S data separately, assessing ITS2 data at the species level and 16S data at the family level. We treated the following variables as binary for all analyses: sample source (burrow vs. surface); rodent exclosure status (rodents present vs. excluded); *Coccidioides* status (positive vs. negative); and pasture of collection (grazed vs. ungrazed pasture).

We estimated alpha diversity for fungal and bacterial taxa using the “phyloseq” package in R [57,60] and compared mean alpha diversity measures (richness, evenness, and Shannon index) across *Coccidioides* status, sample source, rodent exclosure status, and pasture using the Wilcoxon rank-sum test. Additionally, leveraging the paired burrow-surface soil sampling design at each plot, we conducted a Wilcoxon signed-rank test to directly compare the average alpha diversity of replicate precinct samples to that of adjacent replicate surface soil samples, both within and outside of rodent exclosures.

To characterize beta diversity, we transformed ASV sequence data using square-root and Wisconsin double-standardization and then generated a Bray–Curtis dissimilarity matrix using the vegan package [58,61]. We used dimension reduction to visualize the dissimilarity matrices via generating the two principal coordinates explaining the most variation between samples. Finally, we conducted a nested PerMANOVA to see whether microbial community composition differed significantly based on any experimental variables in the dataset [62], adjusting for multiple comparisons using false discovery rate correction [63].

We used checkerboard score analysis, in which counts of pairwise taxa co-occurrences are compared to null expectations based on permuting taxa counts across samples, to examine pairwise taxa associations with *Coccidioides* using the R package “ecospat” version 4.0.0 [59,64]. This analysis is used to determine whether two taxa occur together in a sample more or less often than would be expected by chance. The observed checkerboard scores (C-scores) for each pair of taxa were generated from a presence/absence sample-by-taxa data matrix. We conducted 1000 permutations under a fixed-equiprobable null model, where column (taxa) sums are fixed but sample labels are randomly shuffled, generating a distribution of expected C-scores under the null hypothesis. Finally, we compared the mean expected C-score to the observed C-score to generate a standardized effect score (SES) for each pair of taxa, as well as a significance level, which was adjusted (false discovery rate correction) to account for the number of comparisons [63]. A positive SES indicates that two species co-occurred less often than expected under the null, and a negative SES indicates that they occurred more often than expected. All statistical analyses were conducted in R version 4.3.0.

## 3. Results

We analyzed a total of 318 soil samples (Table S1) via 16S and ITS2 sequencing to investigate the bacterial and fungal communities and their relationship to *Coccidioides immitis* presence. Based on qPCR analysis, 11.9% (38/318) of all soil samples were positive for *Coccidioides* spp. with substantially higher positivity rates for samples from rodent burrows (21.4%, 34/159) compared to surface soils (2.5%, 4/159). Within the *Coccidioides*-positive rodent burrow samples, 67.6% (23/34) were from burrows with active rodent presence, while 32.3% (11/34) were from inactive burrows.

Sequence read depth and other metrics are detailed in Supplementary Text S3 and Figure S1. Within fungal taxa identified to the species level, 65.98% of the fungal species in the dataset belonged to the phylum Ascomycota, 22.74% to Basidiomycota, 4.14% to Mucoromycota, and the remainder to Chytridiomycota, Glomeromycota, Mortierellomycota, and Olpidiomycota. *Coccidioides*-positive samples generally had a higher proportion of Ascomycota than *Coccidioides*-negative samples, and a lower proportion of Basidiomycota (Figure S2). Within bacterial taxa identified to the family level, 39 phyla were represented, and the three phyla containing the most taxa were Actinobacteriota, Bacteroidota, and Proteobacteria (Figure S3).

### 3.1. Alpha Diversity

**Figure 3:**
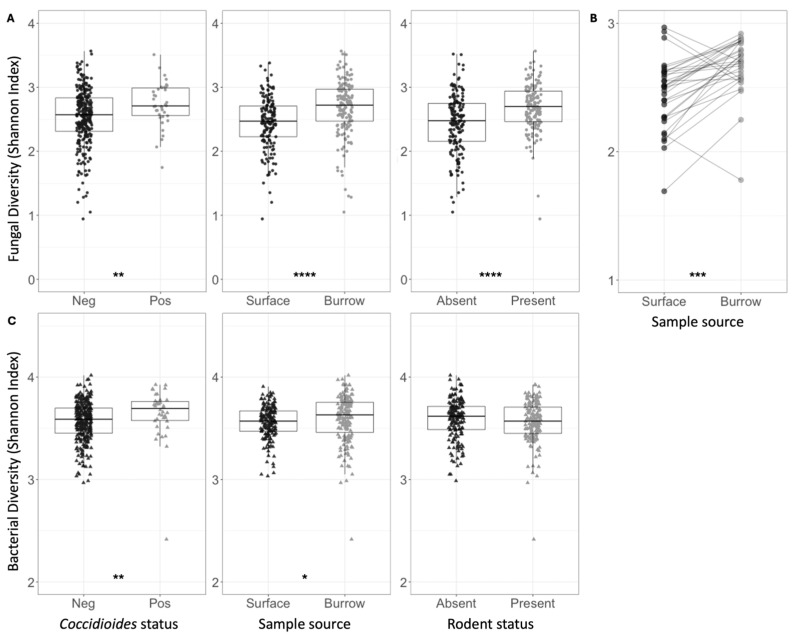
(**A**) Shannon indices for fungal species diversity of all soil samples, grouped and colored based on *Coccidioides* status, surface versus burrow status (regardless of rodent presence), or rodent status. Each circle represents one soil sample. (**B**) Shannon indices for fungal species diversity of soil samples, averaged across five replicate samples collected at each plot. Average estimates for surface soil samples are plotted on the left and burrow soil samples on the right. Lines are drawn to connect spatially paired samples to each other. (**C**) Shannon indices for bacterial family diversity of all soil samples, grouped and colored based on *Coccidioides* status, surface versus burrow status (regardless of rodent presence), or rodent status. Each triangle represents one soil sample. Stars indicate degree of significance based on a Wilcox test such that * = *p* ≤ 0.05, ** = *p* ≤ 0.01, *** = *p* ≤ 0.001, and **** = *p* ≤ 0.0001. No stars indicate *p* > 0.05.

**Figure 4:**
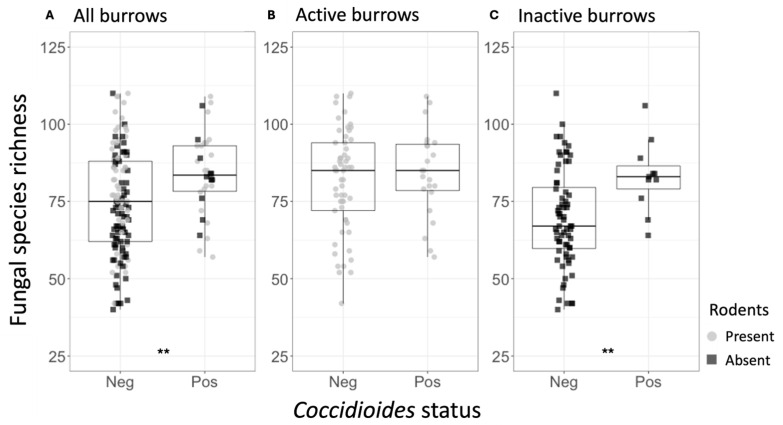
(**A**) Fungal species richness for the rodent burrow samples, separated by *Coccidioides* status (Neg = *Coccidioides*-negative; Pos = *Coccidioides*-positive). Points represent individual samples and are color-coded based on whether they were taken from active (gray) or inactive (black) rodent burrows. (**B**) Fungal species richness for the active rodent burrow samples, separated by *Coccidioides* status. (**C**) Fungal species richness for the inactive rodent burrow samples, separated by *Coccidioides* status. Stars indicate degree of significance based on a Wilcox test such that ** = *p* ≤ 0.01. No stars indicate *p* > 0.05.

Across all samples, the mean fungal species richness and overall fungal diversity (Shannon index) were significantly higher in *Coccidioides*-positive soils (richness = 81.58) compared to negative soils (richness = 63.70, *p* < 0.0001) (Table 1, Figure 3A). Additionally, mean bacterial family richness, evenness, and diversity were significantly higher in *Coccidioides*-positive (richness = 141.68) compared to negative soils (richness = 136.50, *p* < 0.05) (Table 2, Figure 3C). Since most *Coccidioides*-positive samples were also burrow-associated, we further analyzed only soil samples from burrows and found that mean fungal species richness was significantly higher in the *Coccidioides*-positive burrow samples (83.88) than the negative burrow samples (75.06, *p* < 0.01; Figure 4A, Table 1). This difference was driven by burrow samples from which rodents had been excluded. In the burrow samples with active rodent presence, there was no difference in fungal species richness between samples with or without *Coccidioides* presence (Figure 4B). Conversely, in the burrow samples from where rodents were excluded, fungal species richness was significantly higher in samples with *Coccidioides* (83.1) versus without (69.6, *p*-value < 0.01; Figure 4C). Similar trends were observed for bacterial diversity, with *Coccidioides*-positive burrow samples having higher taxa richness than the *Coccidioides*-negative soils, although this was additionally observed when stratifying by burrows with rodent presence and exclusion. However, these bacterial associations were non-significant (Figure S4).

Mean fungal species richness was significantly higher in rodent burrow soils (76.94) compared to surface soils (54.74, *p* < 0.0001), and in soils with active rodent presence (70.42) compared to burrows inside rodent exclosures (61.26, *p* < 0.0001) (Table 1). Fungal species richness was significantly higher in burrow samples compared to surface samples both within (71.48 vs. 51.18, *p* < 0.0001) and outside (82.34 vs. 58.34, *p* < 0.0001) of rodent exclosures. These patterns were recapitulated for fungal species diversity (Shannon index) (Figure 3A and Figure S5, Table 1). Fungal species evenness was significantly higher in soils with rodents present (0.64) versus excluded (0.59, *p* < 0.0001). Comparing paired burrow-surface soil samples at each plot (Figure 2C), fungal species richness and overall alpha diversity (Shannon index) were higher in burrow soils compared to paired surface soils (*p* < 0.001 for Shannon index) (Figure 3B). There were no differences in fungal species evenness across paired samples and no trends in bacterial family richness, evenness, or diversity (Shannon) between paired burrow and surface soils. Despite differences in soil type and cattle grazing, microbial diversity was similar across both pastures (Table 1 and Table 2).

Bacterial family diversity was higher, on average, in burrow soils versus surface soils (Figure 3C, Table 2). When conditioning on rodent exclosure status, bacterial evenness and diversity remained significantly higher in burrow samples (Shannon index: 3.63) compared to surface soil samples within the exclosure (3.55, *p* < 0.001), but the same pattern was not observed outside the exclosure. Bacterial diversity metrics did not vary between samples where rodents were present versus excluded (Figure 3C). Average observed bacterial richness did not vary based on rodent exclosure status or burrow vs. surface soils (Table 2).

### 3.2. Beta Diversity

**Figure 5:**
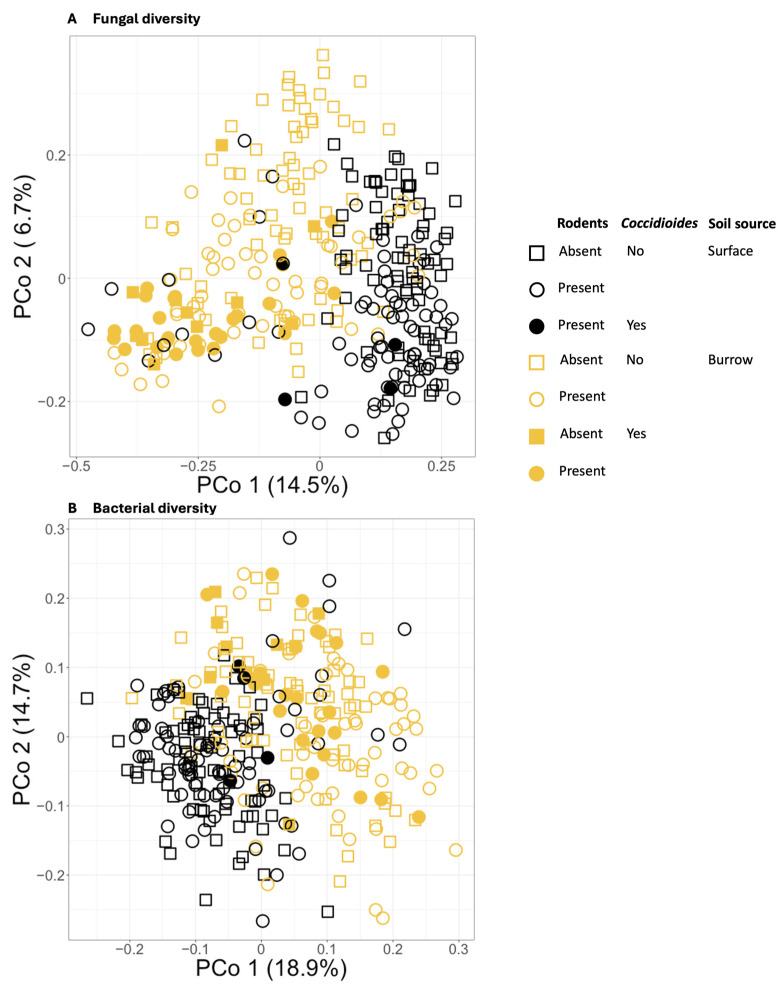
(**A**) Principal coordinates analysis (PCoA) plot showing Bray–Curtis dissimilarities for the soil fungal communities of all samples. (**B**) PCoA plot showing Bray–Curtis dissimilarities for the soil bacterial communities of all samples. Samples drawn from burrows are plotted in orange, and those from the ground surface are plotted in black. Circles represent samples taken from areas with a rodent presence, and squares represent samples taken from rodent exclosures. *Coccidioides*-positive samples are represented by filled shapes, while negative samples are empty shapes.

Both fungal and bacterial beta diversity varied significantly among samples based on all independent variables, including *Coccidioides* status, sample source, and exclosure status, and pasture (Tables S2 and S3). Of these factors, sample source (burrow vs. surface) explained the largest proportion of variation in microbial community composition (for fungi R^2^ = 7%; for bacteria R^2^ = 9%), followed by pasture (for fungi R^2^ = 3%; for bacteria R^2^ = 2%), exclosure status (for fungi R^2^ = 3%; for bacteria R^2^ = 1%), and *Coccidioides* status (for fungi R^2^ = 1%; for bacteria R^2^ = 1%; all *p* < 0.01) (Figure 5). Interactions between variables explained an additional 4% of variation for fungi and 3% of variation for bacteria. Visualization via PCoA showed that samples cluster distinctly based on sample source and, within the fungal dataset only, *Coccidioides* status (Figure 5A).

### 3.3. Co-Occurrence Analysis

**Figure 6:**
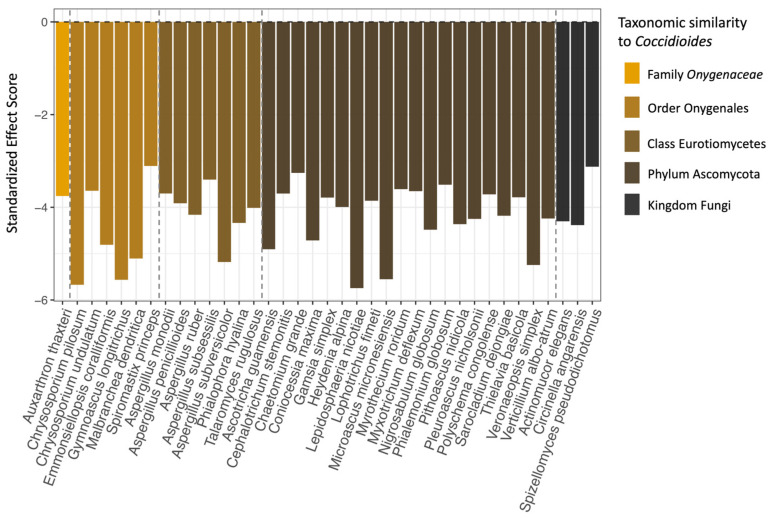
The 37 fungal species that were found to significantly positively co-occur with *Coccidioides* (*x*-axis), as well as their standardized effect scores (*y*-axis). Species are color-coded and ordered based on taxonomic similarity to *Coccidioides*, with dashed lines separating the fungal species in the same family, order, class, and phylum as *Coccidioides*. The fungal species that are most similar are on the far left of the plot and least similar on the far right. Note that groupings are based solely on species inclusion within taxonomic levels, not on direct phylogenetic analysis of the sequences generated for this project.

Based on the checkerboard analysis, 37 species of fungi (6.95% of the full fungal dataset) co-occurred with *Coccidioides* significantly more often than would be expected by chance (Figure 6). No fungal species in our dataset co-occurred with *Coccidioides* less frequently than expected, and no bacterial families had a significant co-occurrence pattern with *Coccidioides* in either direction. Members of the class Eurotiomycetes, order Onygenales, and family *Onygenaceae*, to which *Coccidioides* spp. belongs, were all overrepresented in the set of significantly co-occurring fungal taxa compared to the full dataset (Eurotiomycetes: 37.84% vs. 15.23% in the full dataset; Onygenales: 18.92% vs. 6.2%; and *Onygenaceae*: 2.7% vs. 1.69%). Notably, one species that was identified via our co-occurrence analysis, *Aspergillus penicillioides*, was identified in another study as a potential indicator species for *Coccidioides* [32]. The full taxonomic classifications for all fungal species that had significant, positive co-occurrence patterns with *Coccidioides* are listed in Table S4, along with their standardized effect scores and adjusted *p*-values.

## 4. Discussion

Our study identified several key patterns in soil microbial community dynamics of relevance to the presence of *C. immitis* in the environment. We found that the largest driver of fungal and bacterial community composition and diversity was the soil microhabitat, namely, whether the soils were derived from within rodent burrows or from the ground surface; that is, microbial communities differed more between soils from the ground surface and rodent burrows—regardless of whether rodents were present—than via any other axis of comparison (i.e., rodent exclosure status, *Coccidioides* status). Notably, rodent burrow samples had consistently higher fungal and bacterial diversity than surface soil samples, and for fungal diversity, this held true both when rodents were present and excluded. We also found that *Coccidioides*-positive soil samples had higher microbial diversity, particularly fungal diversity, than negative soil samples. Further, *Coccidioides* was found at higher rates in rodent burrow samples, compared to surface soils, as reported elsewhere [21,24,25]. Finally, despite prior laboratory evidence demonstrating antagonistic interactions between *Coccidioides* and other soil microbes, we found that no fungal or bacterial species showed negative co-occurrence patterns with *Coccidioides*, while 37 fungal species showed positive co-occurrence patterns.

### 4.1. The Soil Microhabitat Drives Patterns in Microbial Diversity

There may be several explanations for the differences in microbial diversity between rodent burrows (with or without rodents present) and surface soils. Abiotic effects, including the structure of rodent burrows and physicochemical properties of soil, can influence microbial populations. Additionally, biotic effects—in particular, the increased availability of nutrients for fungi inside burrows due to rodent activity—likely play an important role in determining patterns of microbial diversity in the ecosystem we studied.

Previous work has shown that rodents affect the soil microbial community through structural creation of burrows [65]. Our study site, the Carrizo Plain National Monument, is home to the endangered *D. ingens* (giant kangaroo rat), an ecosystem engineer whose prolific burrow creation is associated with increased plant productivity, invertebrate diversity, and an abundance of lizards and squirrels [34]. Burrow creation may also play an important role in establishing habitat suitable for diverse bacteria and fungi [65,66], particularly in desert ecosystems where harsh winds, high temperature fluctuations, and low water availability limit microbial establishment and growth [67]. The burrow structure creates a temperature- and moisture-regulated environment [68] that has a more porous soil structure [69,70], maintaining favorable conditions for microbes [71], including *Coccidioides* [24,25]. Our analysis adds to prior evidence using culture-based techniques that found that *Dipodomys* spp. burrows host a more diverse fungal community than the surrounding surface soil [65].

We found that soil source (i.e., burrow versus surface soil) was a more important driver of microbial community composition than rodent presence (Figure 5), suggesting that the burrow microhabitat, regardless of rodent activity, harbors a unique and diverse microbial community in xeric ecosystems. However, we also found that soils from active rodent burrows contained more fungal diversity than soils from inactive burrows, highlighting the important role that rodents play in the ecosystem beyond structural alteration of soil (Table 1). Rodents may additionally affect the soil microbial community via food caching, quarantining their dead, urination, and defecation [66,72]. Rodents in the genus *Dipodomys* are known to utilize their burrows and tunnels for various purposes, including nest creation, food caching, temporary shelter construction, and water drainage [73,74], suggesting that burrow entrances may have variable nutrient availability for microbial life based on rodent usage history. Rodent herbivory of plants may also indirectly affect the soil microbial community. Removal of plants by gophers has been shown to interrupt nitrogen uptake by plants and root-associated microbes, potentially increasing nitrogen availability for other microbes in the soil [75]. Further, it is possible that urea excreted in rodent urine contributes to *Coccidioides* growth in soils. In its host-associated form, *Coccidioides* has been demonstrated to produce urease to break down urea into ammonia, increasing the alkalinity of its environment and promoting the pathogen’s further growth and virulence [76].

### 4.2. Coccidioides Presence Is Associated with Higher Microbial Diversity in Soils

Our results suggest that the presence of *Coccidioides immitis* in the soil is associated with a more diverse soil microbial community as the number of fungal species and bacterial families were both significantly higher in *Coccidioides*-positive soil samples than in negative samples. These findings are aligned with a prior analysis of nine soil samples collected in Venezuela that showed that greater abundance of *C. posadasii* in the soil was associated with greater fungal alpha diversity (Chao1) [31]. Taken together with the increased microbial diversity associated with rodents and burrows, and the 37 fungal species found to significantly co-occur with *Coccidioides*, these findings suggest that favorable microhabitats (e.g., rodent burrows) can facilitate a positive association between soil diversity and *Coccidioides* presence. As the association between *Coccidioides* presence and higher microbial diversity remained even after controlling the soil source and rodent presence, other factors unexplored in this study also likely play a role in determining this relationship.

Among the fungi that significantly co-occurred with *Coccidioides*, those with taxonomic similarity to the pathogen (e.g., same family or order) were overrepresented (Figure 6). This result was surprising as more similar taxa may be expected to occupy similar niches and therefore exhibit greater competition [77]. Evolutionary genomics studies have found that taxa in the Onygenales order have lost the ability to bind cellulose, a plant-based nutrient source [78]. Further, taxa in the *Onygenaceae* family (including *Coccidioides* spp.) have evolved a shared preference for animal-based proteins, such as keratin over plant-based nutrient sources, and therefore may be well adapted to resources found in rodent-associated habitats [78,79,80]. Since these taxa occurred together more frequently than expected in this setting, nutrient availability may not be a limiting factor inside rodent burrows, thereby minimizing the role of antagonistic microbial interactions in shaping *Coccidioides*’ presence in this environment. In addition, rodent burrows may create microclimate conditions to which *Coccidioides* and related taxa are well adapted. Finally, prior work has found that high-temperature and high-salinity conditions in the laboratory stimulate *Coccidioides* growth while inhibiting the growth of microbial antagonists, suggesting that the pathogen may experience fewer competitive interactions in its arid and high-salinity natural environment [30]. Habitat filtering, leading to positive associations between phylogenetically similar taxa based on environmental conditions, has also been observed as a driver of microbial community composition in other systems [81,82].

Our finding of positive associations between *Coccidioides* and related fungal species, as well as an absence of antagonistic associations with any fungal or bacterial taxa, contrasts with prior findings from laboratory experiments. In vitro co-culture challenge assays of *Coccidioides* with bacteria and fungi isolated from soil at *Coccidioides*-positive sites have found that microbial antagonists can inhibit the growth of the fungus in the soil through the secretion of antifungal metabolites [28,29,30]. For instance, several *Streptomyces* spp. and *Bacillus* spp. bacterial strains isolated from soil samples near Bakersfield, California, exhibited antifungal properties towards *C. immitis* and its nonpathogenic relative *Uncinocarpus reesii* in laboratory settings [29]. Similarly, *Bacillus pumilus* and *B. subtilis,* alongside fungal genera *Fennellomyces* spp. and *Ovatospora* spp., were identified as inhibitors of *Coccidioides* after isolation from soils collected in Arizona. [28]. *Ovatospora unipora* and members of the *Streptomycetaceae* and *Bacillaceae* families were present in our datasets but were not associated with *Coccidioides*’ presence or absence. Further, our co-occurrence analysis did not identify any species negatively associated with *Coccidioides*, suggesting these antagonistic interactions may be mitigated in *Coccidioides*’ natural environment.

Collectively, these findings suggest that the microbial co-occurrence patterns with *Coccidioides* observed here, along with trends in microbial diversity, are largely influenced by the rodent burrow microhabitat. We note that our study was observational in nature and caution that these microbial associations are not causally interpretable. It is feasible that other factors are driving the observed microbial co-occurrence patterns. We did not examine soil characteristics such as nutrient availability, pH, or moisture, which likely influence soil fungal and bacterial communities. Experimental validation examining the individual and combined effects of these and other factors on microbial interactions is needed to further elucidate the variables driving microbial co-occurrence patterns. Our study has additional limitations in that the assays we used to detect *Coccidioides* and other fungi and bacteria are unable to distinguish between viable organisms and residual DNA present in the soil. Future studies characterizing the abundance and viability of *Coccidioides* in the soil across seasons, interannual climate trends, nutrient availability, and soil types would help resolve the roles of abiotic versus biotic drivers of soil microbial community diversity and structure.

## 5. Conclusions

Our study provides evidence that the soil microhabitat is critical for determining the relationship between the soil microbial community and the presence of *Coccidioides*. In regions that are highly endemic for the pathogen, small burrowing mammals (including the ecosystem engineer, *D. ingens*) may play a crucial role in altering soil conditions and providing favorable microenvironments for diverse fungal and bacterial taxa, including *Coccidioides* spp. and its relatives in the Onygenales order. Further, we found that the soil microbial community is more diverse in *Coccidioides*-positive soils, even when controlling for other variables, suggesting that additional unmeasured factors may play a role in determining this association. While laboratory studies provide crucial insight into potential microbial interactions between *Coccidioides* and other soil microbial taxa, including mechanistic evidence of antagonistic or competitive relationships, these interspecific interactions may not be borne out in natural settings, thus highlighting the importance of continued environmental sampling to uncover the factors driving *Coccidioides*’ presence in the environment.

## Figures and Tables

**Table 1:** Mean fungal species alpha diversity measures (richness, evenness, and Shannon index) for all soil samples. Overall diversity metrics for the whole sample set are reported, as are metrics for subsets of samples grouped based on the following measured variables: pasture, sample source, rodent exclosure status, and *Coccidioides* status. Additionally, alpha diversity metrics for all burrow samples are reported, stratified based on *Coccidioides* status, and further based on rodent exclosure status.

	Richness Mean (Q1, Q3)	Evenness Mean (Q1, Q3)	Shannon Mean (Q1, Q3)
Overall (*n* = 318)	65.84 (51.25, 80.0)	0.62 (0.57, 0.67)	2.56 (2.34, 2.85)
Grazed pasture (*n* = 158)	67.32 (54.00, 83.25)	0.60 (0.56, 0.66)	2.53 (2.34, 2.81)
Ungrazed pasture (*n* = 160)	64.38 (49.75, 79)	0.63 (0.58, 0.68)	2.59 (2.33, 2.88)
Burrow (*n* = 159)	76.94 (64.50, 89.00)	0.62 (0.58, 0.67)	2.67 (2.47, 2.97)
Surface (*n* = 159)	54.74 (46.00, 61.50)	0.61 (0.56, 0.67)	2.44 (2.23, 2.71)
Rodents absent (*n* = 159)	61.26 (48.50, 71.00)	0.59 (0.54, 0.66)	2.44 (2.16, 2.75)
Rodents present (*n* = 159)	70.42 (54.00, 86.00)	0.64 (0.60, 0.68)	2.68 (2.46, 2.94)
*Coccidioides*-positive (*n* = 38)	81.58 (69.75, 92.25)	0.62 (0.58, 0.67)	2.73 (2.56, 2.99)
*Coccidioides*-negative (*n* = 280)	63.70 (50.00, 75.25)	0.61 (0.57, 0.67)	2.54 (2.31, 2.83)
Burrow (*n* = 159)			
*Coccidioides*-positive (*n* = 34)	83.88 (78.25, 93.00)	0.62 (0.57, 0.67)	2.73 (2.54, 2.99)
Rodents absent (*n* = 11)	83.09 (79.00, 86.50)	0.62 (0.57, 0.68)	2.75 (2.60, 2.98)
Rodents present (*n* = 23)	84.26 (78.50, 93.50)	0.62 (0.58, 0.67)	2.72 (2.53, 2.98)
*Coccidioides*-negative (*n* = 125)	75.06 (62.00, 88.00)	0.62 (0.58, 0.67)	2.66 (2.42, 2.96)
Rodents absent (*n* = 68)	69.60 (59.75, 79.50)	0.61 (0.57, 0.66)	2.57 (2.38, 2.85)
Rodents present (*n* = 57)	81.56 (72.00, 94.00)	0.63 (0.60, 0.68)	2.77 (2.56, 3.02)

**Table 2:** Mean bacterial family alpha diversity measures (richness, evenness, and Shannon index) for all soil samples. Overall diversity metrics for the whole sample set are reported, as are metrics for subsets of samples grouped based on the following measured variables: Pasture of collection, sample source, rodent exclosure status, and *Coccidioides* status.

	Richness Mean (Q1, Q3)	Evenness Mean (Q1, Q3)	Shannon Mean (Q1, Q3)
Overall (*n* = 318)	137.12 (128.00, 150.00)	0.73 (0.71, 0.75)	3.57 (3.47, 3.71)
Grazed pasture (*n* = 158)	137.06 (128.00, 150.00)	0.73 (0.71, 0.75)	3.57 (3.47, 3.70)
Ungrazed pasture (*n* = 160)	137.18 (128.00, 148.25)	0.73 (0.71, 0.75)	3.58 (3.48, 3.72)
Burrow (*n* = 159)	137.38 (128.50, 150.00)	0.73 (0.71, 0.75)	3.59 (3.46, 3.75)
Surface (*n* = 159)	136.86 (128.00, 149.00)	0.72 (0.71, 0.74)	3.56 (3.47, 3.67)
Rodents absent (*n* = 159)	137.36 (128.00, 150.00)	0.73 (0.71, 0.75)	3.59 (3.49, 3.71)
Rodents present (*n* = 159)	136.89 (128.50, 148.00)	0.73 (0.71, 0.75)	3.56 (3.45, 3.71)
*Coccidioides*-positive (*n* = 38)	141.68 (130.25, 159.50)	0.74 (0.72, 0.76)	3.64 (3.58, 3.76)
*Coccidioides*-negative (*n* = 280)	136.50 (128.00, 149.00)	0.73 (0.71, 0.75)	3.57 (3.45, 3.70)

## Data Availability

The sequencing data generated and analyzed during the current study are available on the National Center for Biotechnology Information Sequence Read Archive under the following Accession Numbers: PRJNA1201319 and PRJNA1201328. The associated data tables and code scripts are available at https://github.com/mradosevich/CocciMicrobialCommunities (accessed on 9 April 2025).

## Supplementary Materials

The following supporting information can be downloaded at https://www.mdpi.com/article/10.3390/jof11040309/s1: Text S1. Materials and Methods, Soil DNA Extraction; Text S2. Materials and Methods, Quantitative PCR to detect Coccidioides spp.; Text S3. Results; Figure S1. Rarefaction curves for (A) ITS2 and (B) 16S sequencing. Each line denotes a single sequenced sample. Note, X and Y axes differ between plots; Figure S2. Stacked bar plot showing the proportion of fungal species belonging to each phylum for the full sample set and within sample subgroups. The seven bars are, from the left to right, all samples, *Coccidioides*-positive samples from burrows where rodents are present, from burrows where rodents are absent, and from surface soils; and *Coccidioides*-negative samples from burrows where rodents are present, from burrows where rodents are absent, and from surface soils; Figure S3. Stacked bar plot showing the proportion of bacterial families belonging to each phylum for the full sample set and within sample subgroups. The seven bars are, from the left to right, all samples, *Coccidioides*-positive samples from burrows where rodents are present, from burrows where rodents are absent, and from surface soils; and *Coccidioides*-negative samples from burrows where rodents are present, from burrows where rodents are absent, and from surface soils; Figure S4. (A) Bacterial family richness for the rodent burrow samples, separated by *Coccidioides* status (Neg = *Coccidioides*-negative, Pos = *Coccidioides*-positive). Points represent individual samples and are color-coded based on whether they were taken from active (gray) or inactive (black) rodent burrows. (B) Bacterial family richness for the active rodent burrow samples, separated by *Coccidioides* status. (C) Bacterial family richness for the inactive rodent burrow samples, separated by *Coccidioides* status. Stars indicate degree of significance based on a Wilcox test, such that * = *p* ≤ 0.05, ** = *p* ≤ 0.01, *** = *p* ≤ 0.001, and **** = *p* ≤ 0.0001. No stars indicate *p* > 0.05; Figure S5. (A) Fungal species diversity for samples taken from the rodent exclosure (where rodents are absent), separated based on soil source (surface or burrow). Points represent individual samples. Samples taken from the ground surface are in black, and those taken from inside rodent burrows are in grey. (B) Fungal species diversity for samples taken from the non-exclosure (where rodents are present), separated based on soil source (surface or burrow). Stars indicate degree of significance, such that * = *p* ≤ 0.05, ** = *p* ≤ 0.01, *** = *p* ≤ 0.001, and **** = *p* ≤ 0.0001. No stars indicate *p* > 0.05; Table S1. Distribution of samples (n = 318) based on sample characteristics, including pasture, rodent status (rodents absent or present), sample source (burrow or surface), and *Coccidioides* status (positive or negative); Table S2. Nested PerMANOVA model outputs for the ITS2 dissimilarity matrix, including sum of squares, R^2^, pseudo-F statistic, and *p*-values for all individual variables and interaction terms; Table S3. Nested PerMANOVA model outputs for the 16S dissimilarity matrix, including sum of squares, R^2^, pseudo-F statistic, and *p*-values for all individual variables and interaction terms; Table S4. Full taxonomic classifications of the fungal species identified to significantly positively co-occur with *Coccidioides.* The classification for *Coccidioides immitis* is listed in the top row for reference. Orange shading denotes the taxonomic classification that *Coccidioides* is within. Standardized effect scores and adjusted *p*-values are listed for each species.
