# Supplementary material for: Characterizing the Soil Microbial Community Associated with the Fungal Pathogen Coccidioides immitis
Source: J Fungi (Basel). 2025 Apr 14;11(4):309. doi: 10.3390/jof11040309 (PMC12028473; doi:10.3390/jof11040309)
Supplement: Supplementary file 1 [file jof-11-00309-s001.zip › supp_text_JoF.pdf]

Supplementary text for: Characterizing the soil microbial community associated with the fungal pathogen *Coccidioides immitis*

Molly Radosevich[1], Jennifer Head[2,3], Lisa Couper[1], Amanda Weaver[1], Simon Camponuri[1], Liliam Montoya[4], John Taylor[4], and Justin Remais[1,*]

1 Environmental Health Sciences, University of California Berkeley, Berkeley, California, 94720
2 Department of Epidemiology, University of Michigan, Ann Arbor, Michigan, 48109
3 Institute of Global Change Biology, University of Michigan, Ann Arbor, Michigan, 48109
4 Plant and Microbial Biology, University of California Berkeley, Berkeley, California 94720
* Correspondence: jvr@berkeley.edu

Supplementary Text S1

MATERIALS AND METHODS

Soil DNA extraction

To extract genomic DNA from each sample, we transferred approximately 250 mg of soil into a Lysing Matrix E tube prefilled with a mixture of ceramic, silica, and glass beads (MPBio, Burlingame, California, USA) and lysis buffer. Samples were homogenized using a bead beater (MP Bio FastPrep-24 5G Cell Disruptor) for two cycles of 30 seconds each at six meters/second, separated by a five-minute rest period. Then, we centrifuged samples and transferred the supernatant to a clean 2-mL tube. Past this point, we conducted the remainder of the protocol as specified by the DNeasy PowerSoil Pro Kit manual on the laboratory benchtop.

Supplementary Text S2

MATERIALS AND METHODS

Quantitative PCR to detect *Coccidioides* spp.

Quantitative PCR was conducted following the reagent concentrations and thermocycler conditions detailed in the CoccciENV assay [1], which has become the gold standard for environmental detection of *Coccidioides* in soils. We used a Taqman probe targeting a highly specific copia-like retrotransposon with repeated copies in the *Coccidioides* genome, and a primer mixture containing 11 forward and 18 reverse primers to encompass many alleles of the target region. Samples were run on a 96-well plate in 20 μL per reaction, with each reaction containing 10 μL of Taqman Environmental Master Mix 2.0 (Thermo Fisher Scientific, Waltham, Massachusetts, USA), 2 μL of an assay mix containing forward and reverse primers (Integrated DNA Technologies, Coralville, Iowa, USA) and a Taqman probe (Thermo Fisher Scientific, Waltham, Massachusetts, USA), and 8 μL template DNA. On each plate, we ran genomic DNA from a clinical isolate of *Coccidioides posadasii*, the Silviera strain, as a positive control, and a non-template control with water as a negative control.

Supplementary Text S3

RESULTS

We obtained a total of 24,053,261 reads from ITS2 sequencing and 25,709,195 from 16S sequencing, with 532 ASVs matched to known fungal species and 394 ASVs matched to known bacterial families. Within the known taxa, ITS2 sequencing returned a total of 9,731,837 reads (mean number of reads per sample 30,603), while 16S sequencing returned a total of 12,197,453 reads (mean number of reads per sample 38,357). Rarefaction curves indicated that all samples obtained sufficient sequencing depth (Figure S1). Positive controls amplified as expected, and no amplification was observed in negative controls.

REFERENCES

[1] J.R. Bowers, K.L. Parise, E.J. Kelley, D. Lemmer, J.M. Schupp, E.M. Driebe, D.M. Engelthaler, P. Keim, B.M. Barker, Direct detection of *Coccidioides* from Arizona soils using CocciENV, a highly sensitive and specific real-time PCR assay, Med. Mycol. 57 (2019) 246–255. https://doi.org/10.1093/mmy/myy007.
