# Supplementary figures and images for: Characterizing the Soil Microbial Community Associated with the Fungal Pathogen Coccidioides immitis
Source: J Fungi (Basel). 2025 Apr 14;11(4):309. doi: 10.3390/jof11040309 (PMC12028473; doi:10.3390/jof11040309)

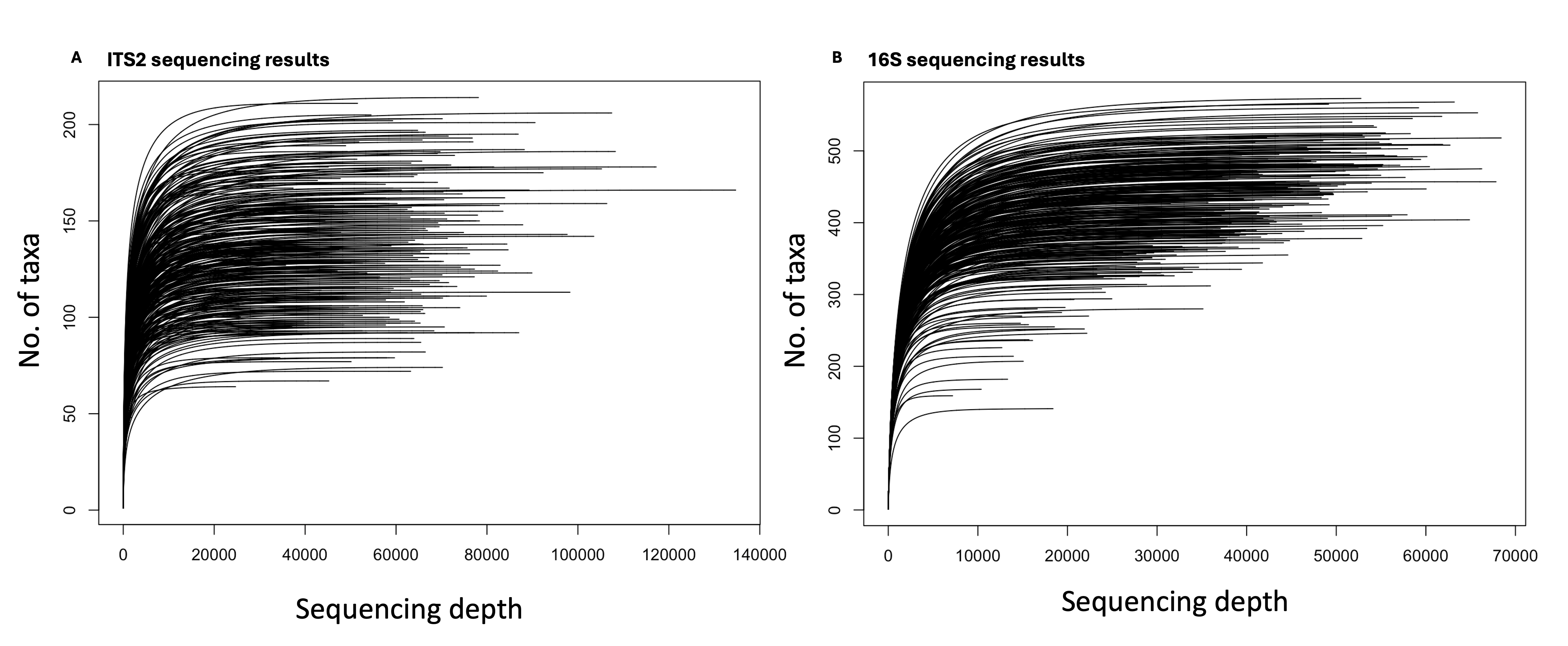

Supplement: Supplementary file 1 [file jof-11-00309-s001.zip › fig_S1.png]

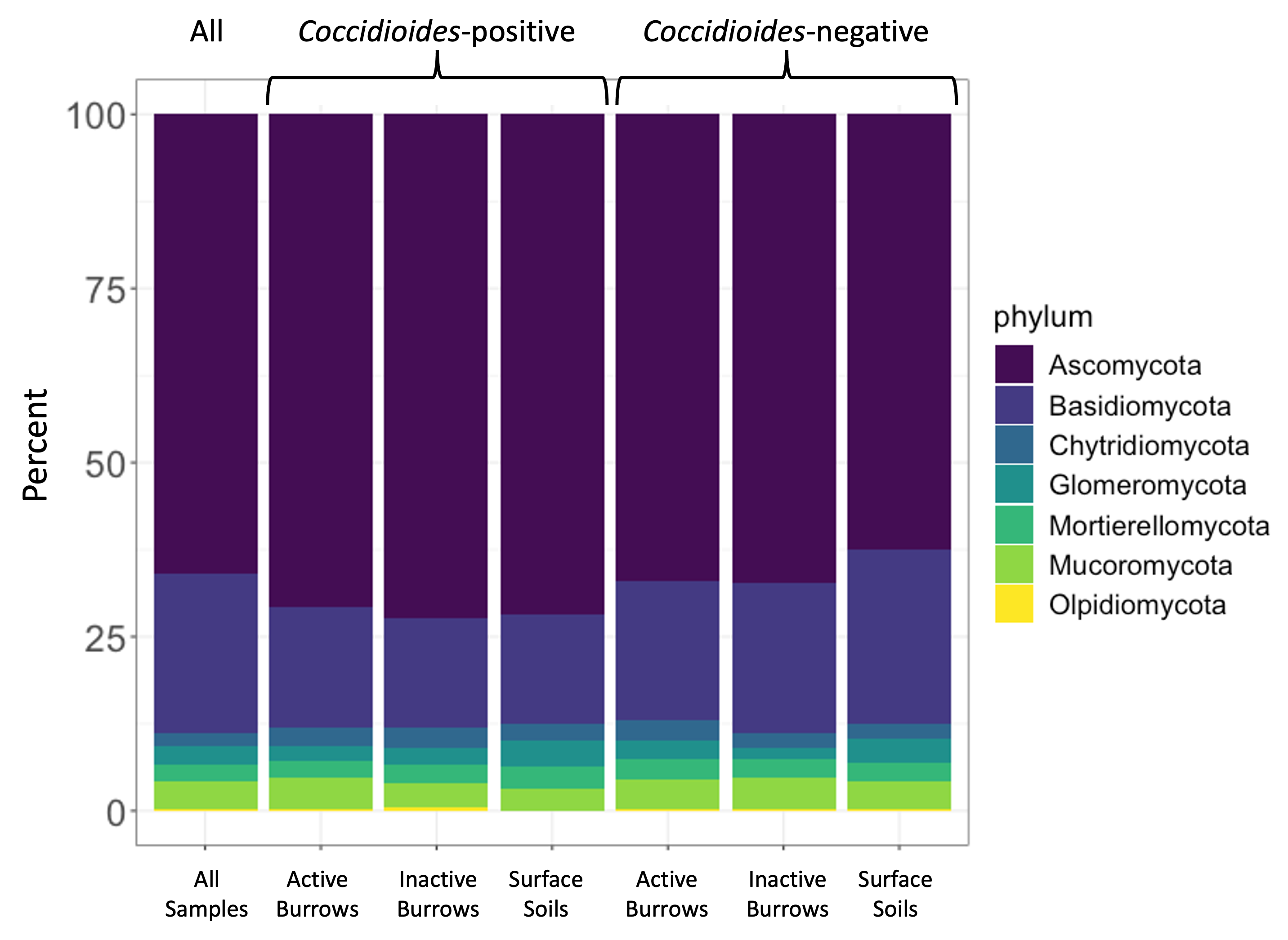

Supplement: Supplementary file 1 [file jof-11-00309-s001.zip › fig_S2.png]

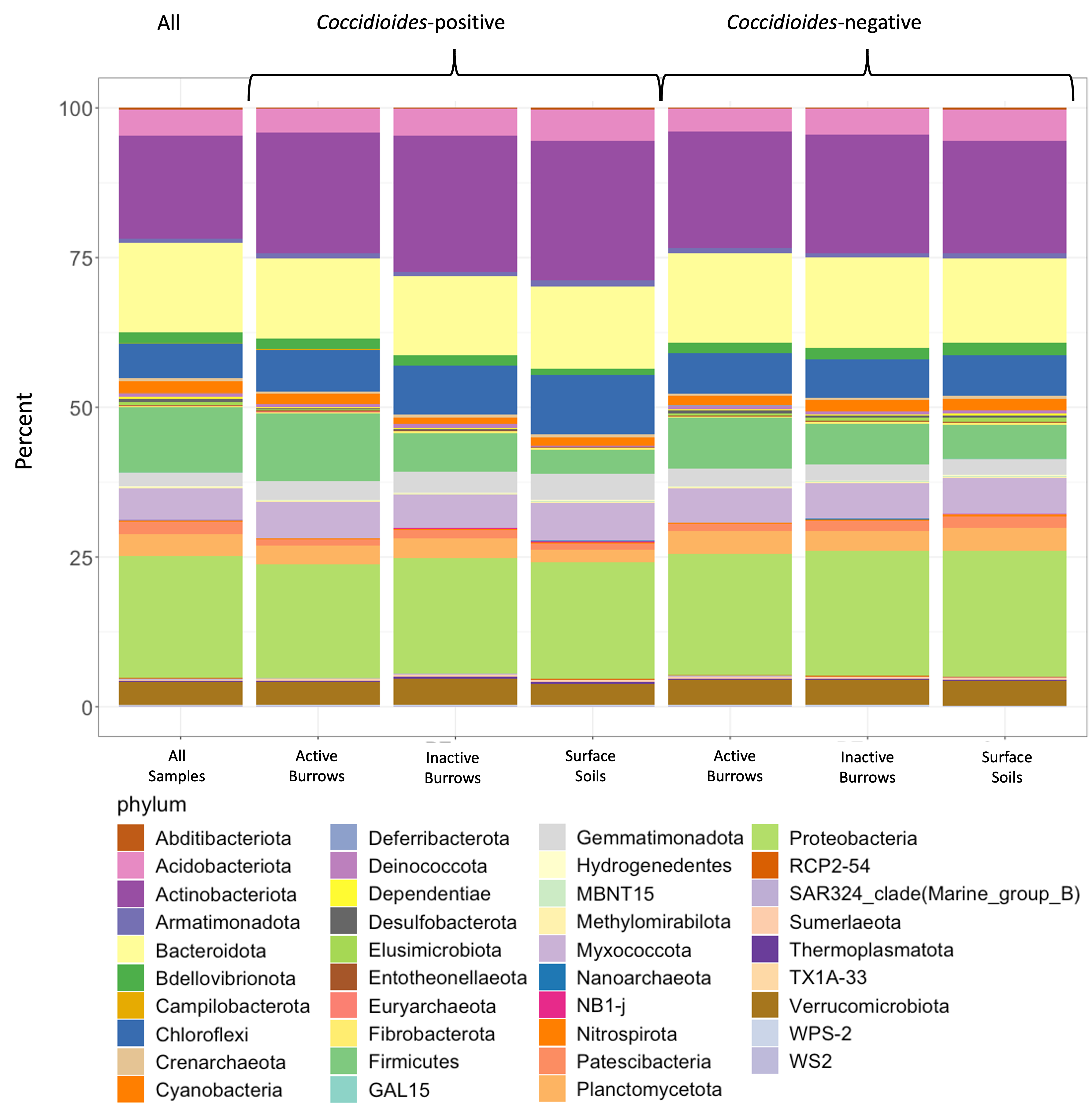

Supplement: Supplementary file 1 [file jof-11-00309-s001.zip › fig_S3.png]

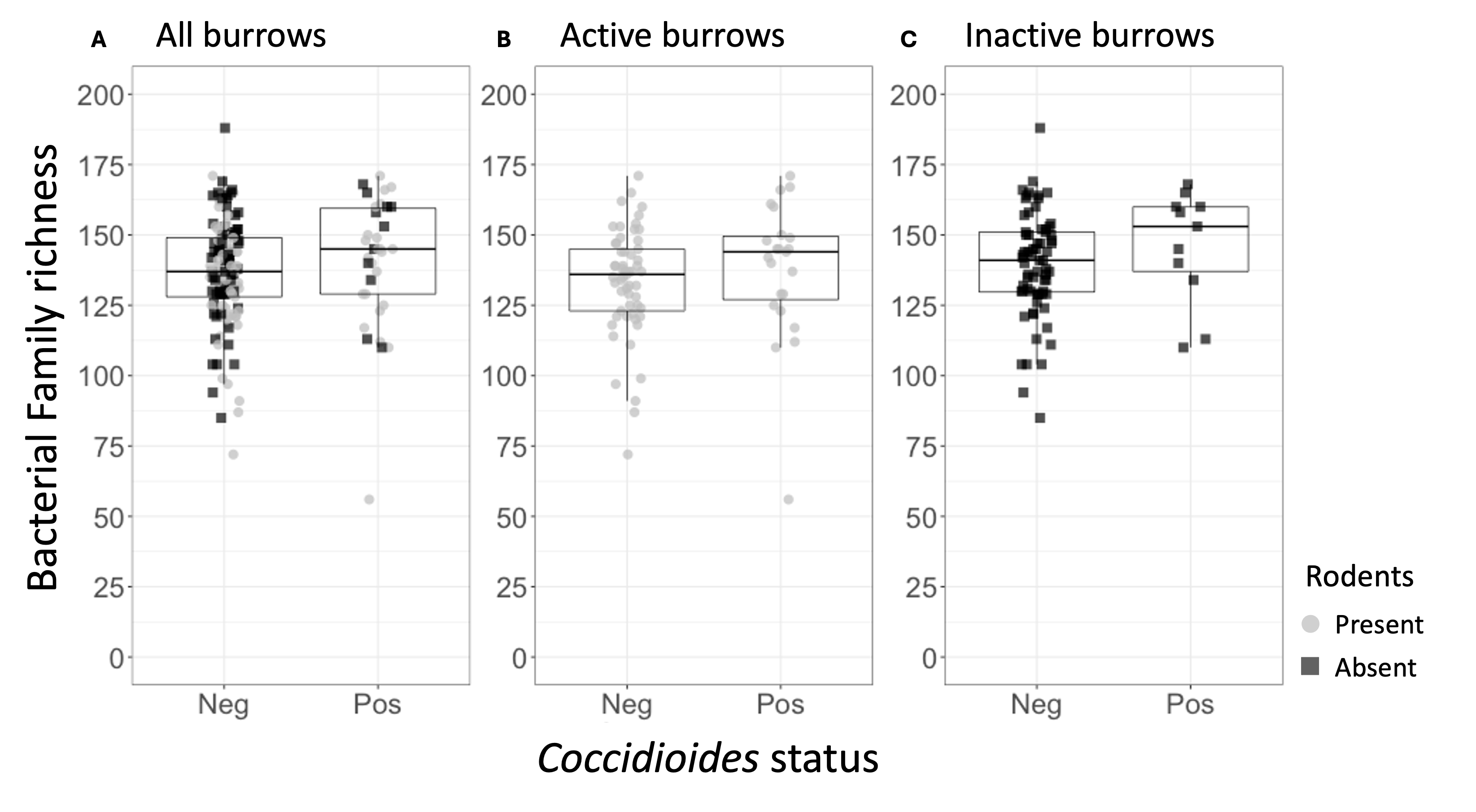

Supplement: Supplementary file 1 [file jof-11-00309-s001.zip › fig_S4.png]

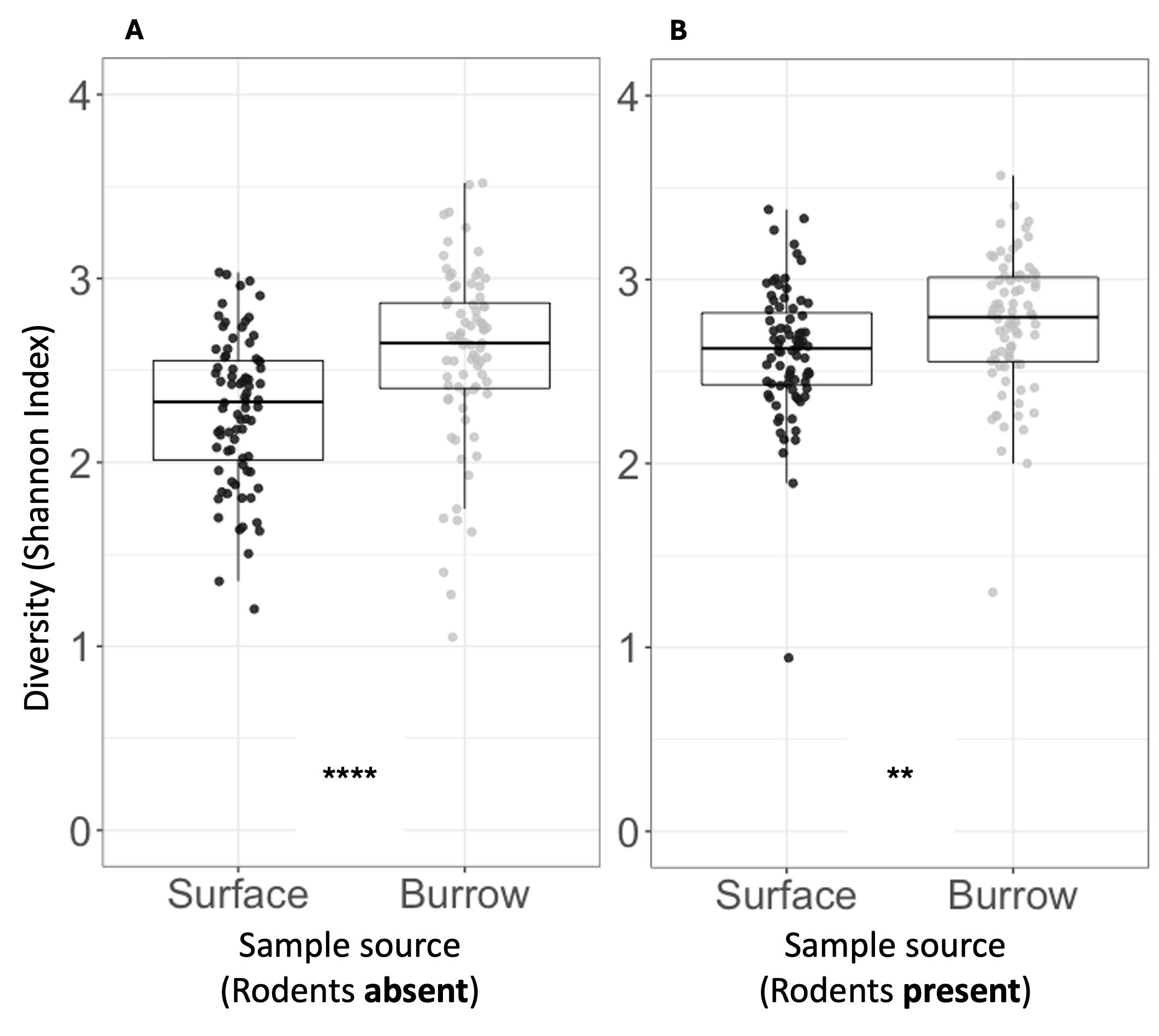

Supplement: Supplementary file 1 [file jof-11-00309-s001.zip › fig_S5.png]
